# CycleStyleGAN-Based Knowledge Transfer for a Machining Digital Twin

**DOI:** 10.3389/frai.2021.767451

**Published:** 2021-11-25

**Authors:** Evgeny Zotov, Visakan Kadirkamanathan

**Affiliations:** Department of Automatic Control and Systems Engineering, University of Sheffield, Sheffield, United Kingdom

**Keywords:** knowledge transfer, transfer learning, domain adaptation, incremental learning, artificial intelligence, deep learning, generative adversarial network, industry 4.0

## Abstract

Digitalisation of manufacturing is a crucial component of the Industry 4.0 transformation. The digital twin is an important tool for enabling real-time digital access to precise information about physical systems and for supporting process optimisation *via* the translation of the associated big data into actionable insights. Although a variety of frameworks and conceptual models addressing the requirements and advantages of digital twins has been suggested in the academic literature, their implementation has received less attention. The work presented in this paper aims to make a proposition that considers the novel challenges introduced for data analysis in the presence of heterogeneous and dynamic cyber-physical systems in Industry 4.0. The proposed approach defines a digital twin simulation tool that captures the dynamics of a machining vibration signal from a source model and adapts them to a given target environment. This constitutes a flexible approach to knowledge extraction from the existing manufacturing simulation models, as information from both physics-based and data-driven solutions can be elicited this way. Therefore, an opportunity to reuse the costly established systems is made available to the manufacturing businesses, and the paper presents a process optimisation framework for such use case. The proposed approach is implemented as a domain adaptation algorithm based on the generative adversarial network model. The novel CycleStyleGAN architecture extends the CycleGAN model with a style-based signal encoding. The implemented model is validated in an experimental scenario that aims to replicate a real-world manufacturing knowledge transfer problem. The experiment shows that the transferred information enables the reduction of the required target domain data by one order of magnitude.

## 1 Introduction

The digital twin is a precise representation of a physical object or process within the digital realm. The definition of this concept, initially conceived within the aerospace industry (Shafto et al., 2010), has evolved to encompass whole ecosystems that are recreated digitally as cyber-physical systems (CPS) (Bajaj et al., 2016). Modern understanding of the underlying system characteristics is highly influenced by the introduction of the big data and the industrial internet of things (IIoT) solutions (Lee et al., 2013). Thus, when approached within the scope of the transition towards Industry 4.0, the development of the digital twin requires a holistic approach to data acquisition, modelling and analysis, as multiple interconnected components need to be assembled to fully deliver the value a digital twin is expected to produce as a decision-making tool (Grieves and Vickers, 2017; Negri et al., 2017). Before such a holistic vision can be realised in practice, the research and industrial communities would have to present innovation in several fields, including infrastructure, process monitoring and predictive systems. The unification of the physical and the digital data from across the various steps of the object’s life cycle introduces novel difficulties that challenge the established analysis methods (Tao et al., 2018).

Simulation modelling is one of the key components of the digital twin. It is widely used in the verification and evaluation of engineering systems and their performance and functionality. In manufacturing the virtual recreation of production enables the acquisition of insights into the behaviour of the product. A product’s features can be analysed both online and offline and their characteristics predicted prior to the end of the manufacturing process (Papananias et al., 2019a).

The increase of the computational efficiency provided by the improvements to the software and hardware solutions over the recent years significantly lowered the barriers that previously limited the practical applicability of simulations (Smith and Tlusty, 1991). The machining domain steadily increases its reliance in simulation modelling for analysis of tool stress (Özel and Altan, 2000), forces (Afazov et al., 2010), surface finish (Campomanes and Altintas, 2003), machining stability (Altintas and Weck, 2004) and verification of the physics-based models (Altintas et al., 2014; Thepsonthi and Özel, 2015; Shetty et al., 2017; Greis et al., 2020).

The diversity of machining error causes, as well as their dynamic character, substantially distort model predictions, posing a major problem that continues to be extensively investigated by manufacturing researchers (Wilhelm et al., 2001; Monostori, 2003; Elmaraghy et al., 2012; Li et al., 2015; Tidriri et al., 2016). Material uncertainties (e.g., its differences from the specification) and machining uncertainties (e.g., the thermal errors) are among the many of the error causes. These production-process-related uncertainties are transmitted and amplified through measurement errors originating from software faults, instrumental errors, fixturing errors, etc., (Forbes, 2013; Papananias et al., 2019a). The result is the chaotic variability of the behaviour of the modelled processes under different conditions and in heterogeneous environments.

The use of physics-based modelling approaches, while oftentimes is theoretically able to produce highly accurate results, becomes practically limited, considering the diversity and complexity associated with the scale of Industry 4.0 environments. The reasons for this limitation stem from the dynamic character of the flow of the environmental properties (Niggemann et al., 2015) and the fact that the physics-based models have to be reconfigured for the new environments, often requiring manual intervention. The automation of the modelling process attainable with data-driven modelling circumvents this issue, aligning the states of a physical object and its digital twin. Thus, while physics-based simulations are successful at machining abnormalities prediction, data-driven approaches enable higher adaptability to the dynamic internal and environmental conditions (Friedrich et al., 2018).

The main limitation of the data-driven modelling is its requirement for data. A data-driven model would not be able to reason from first principles unless merged into a hybrid system with the incorporation of a physics-based model. Such hybrid approaches are actively investigated by the research community (Greis et al., 2020), but their implementation is limited by the access to the underlying models of the physics-based tools used in industry. The proprietary modelling software rarely provides flexible integration access to its outputs, even less so to its internal procedures. Nevertheless, the information contained within such models is of great value, usually reflected in the price tag of the proprietary modelling software. The extraction of this information in a reusable form is one of the applications of the methods found in the knowledge transfer research domain and is the main topic of this paper.

Knowledge transfer for data analytics deals with the problem of heterogeneous or non-stationary environments, where a model effective in some environment requires adaptation to new or changed conditions to remain accurate (Bang et al., 2019). In the context of manufacturing this usually implies that the target domain data is very limited, thus the need for efficient knowledge transfer from data-abundant domains. Knowledge transfer is usually regarded as an approach for information transfer between machine learning methods (Pan and Yang, 2010). But in the context of simulation modelling a previous work shows that it is not strictly necessary for the source model to be a learned model, as knowledge is extractable from physics-based simulation models using a generative adversarial network (GAN) (Zotov et al., 2021).

The generative adversarial network is a kind of an artificial neural network (ANN) that is built on a competitive minimax game between two ANNs: the generator, which learns to generate data samples, and the discriminator, which learns to detect the fake data samples amongst the real ones (Goodfellow et al., 2014). As a result, the data distribution generated by the generator network approaches the true distribution of the data which may be directly used for simulation of the underlying process. Among the many extensions to the original GAN architecture, one of the most relevant for the knowledge transfer research is the CycleGAN proposed by (Zhu et al., 2017). Section 2.2 describes this architecture, as well as the StyleGAN (Karras et al., 2018) model that form the base of the method proposed in this paper.

A recent review by Bang et al. (2019) groups the knowledge transfer methods into two groups: incremental learning and domain adaptation. The main difference between the approaches in these groups is the discarding of the source domain data during incremental learning, as only the source domain knowledge encoded *via* a trained model is carried over to the target domain training phase (Giraud-carrier, 2013). Domain adaptation, on the contrary, implies that the source domain data is at least partially available and used to learn a mapping between the source and the target domains (Ganin et al., 2016; Weiss et al., 2016).

Several recent domain adaptation methods are inspired by the adversarial interactions within GANs, as reflected in the terminology used to describe these techniques: adversarial domain adaptation. These are usually categorised as either feature-level (Liu and Tuzel, 2016; Sun et al., 2016) or pixel-level adversarial domain adaptation approaches (Bousmalis et al., 2017; Shrivastava et al., 2017) with some of the recent works merging the two in hybrid adversarial domain adaptation models, e.g. Bousmalis et al. (2018).

The widespread digitalisation within the transition towards Industry 4.0 creates a drive for flexible and efficient data-driven simulation modelling. The existing simulation solutions frequently lack the required flexibility and integration access to be effective in a heterogeneous and dynamic environment of the interconnected cyber-physical systems. Nevertheless, the knowledge contained within these costly models is often valued at a very high price. The efficient use of this knowledge is therefore an important concern for any business employing such simulations.

The work presented here proposes a solution for extraction of the knowledge from the existing manufacturing simulation tools applicable both to physics-based and to data-driven source models, thus enabling business cost optimisation. The proposed approach implements a novel CycleStyleGAN domain adaptation model by introducing the style-based signal representation into the CycleGAN framework. This paper evaluates the effectiveness of the proposed knowledge transfer method and compares it to an incremental learning approach validated under identical conditions. A proposed use case of the developed model for manufacturing process optimisation is also discussed in section 4.

## 2 Materials and Methods

### 2.1 Milling Vibration Datasets

Due to the high cost of collection, manufacturing process data is currently a limited resource. The commercial secrecy of such data adds to the difficulty of using it in public research. Real implementations of data-driven digital twins are thus likely to be trained on existing and established physics-based models and fine-tuned using a combination of simulated and empirical data. The simulation that generated the dataset utilised in this study is a surrogate for a real-world data-generation process. On one hand, because of the complete control over data creation, this enables a thorough examination of the digital twin component performance. On the other hand, the proposed method approximates the real-world case of transitioning from pure physics-based modeling to a scenario including both physics and experimental data.

**FIGURE 1:**
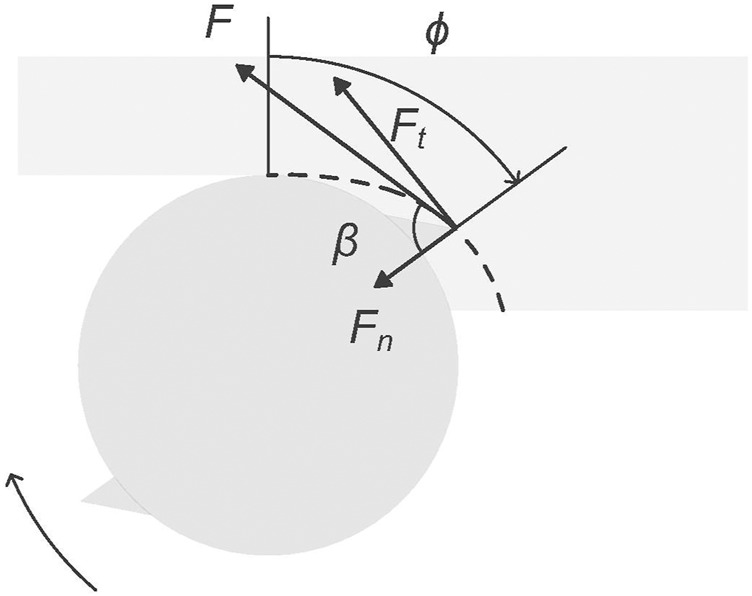
Geometrical representation of the forces simulated by the physics-based model. *F* is the cutting force and *β* is the force angle. *F*_*t*_ and *F*_*n*_ denote the tangential and the normal cutting forces and *ϕ* is the cutting tooth angle.

**FIGURE 2:**
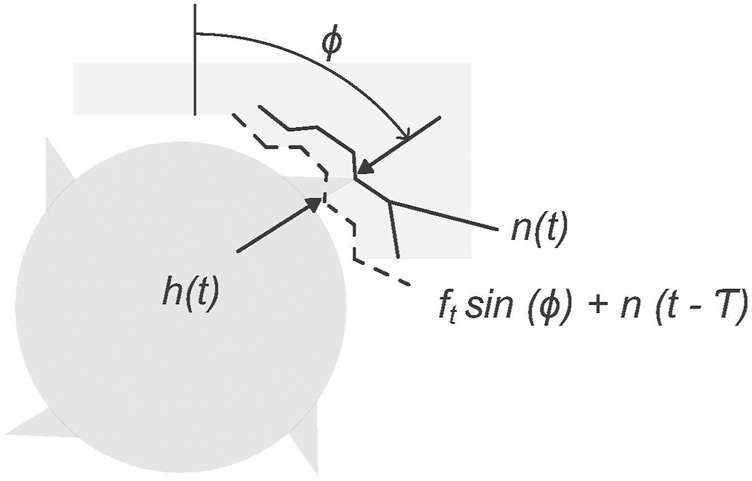
Workpiece geometry produced by the simulation model. *h*(*t*) is the instantaneous depth of cut at time *t* that is the distance between the current normal direction vibration level at angle *ϕ* and the cut surface at angle *ϕ* produced at time *t* − *T*, where *T* is the time period of cutting tool revolution between two neighbouring cutting teeth.

The GAN models used in this study are trained using synthetic datasets generated by a physics-based time-domain simulation model based on Schmitz and Smith (2019). The simulation iteratively determines the forces generated by the interaction of a non-rigid machining tool’s cutting teeth with a rigid workpiece (Figure 1). These forces are utilised to calculate the cutting tool’s acceleration, velocity, and displacement, i.e. vibration. Vibration is chosen as the analysed signal type because of its low anticipated collection cost and its use in machining process analysis. To determine which cutting teeth are in the cut at each time step, the simulation records the orientation of each cutting tooth and the workpiece contour generated by material removal (Figure 2). The process considered in this work is a linear non-slotting milling cut on a metal workpiece using a straight-teeth cutting tool.

The physics-based model accepts several variables that control the deterministic simulation, including machining parameters that can be controlled during the metal cutting process configuration and parameters that are dependent on the workpiece material, machining tool, and the manufactured product characteristics. Three datasets are created for the experiments presented in this paper. Dataset 1 represents source domain data. Datasets 2 and 3 correspond to the target domains that differ from the source domain, respectively, either slightly or significantly. The similarity between dataset 2 and dataset 1 portrays a situation of a small difference in the environment temperature or the machined material properties between the two domains. Dataset 3 represents a case of substantially varied properties of the underlying signals, for example resulting from a change of machining tool.

Table 1 details the process simulation variables, followed by the values used to generate the training data. The hyphens indicate the values used in datasets 2 and 3 that are unchanged as compared to dataset 1. Chip width and spindle speed, which range from 0.004 to 0.005 mm and 3,000 to 4,000 rpm respectively, are the characteristics that vary among the samples in a generated dataset. The produced signals reflect the cutting tool’s displacement in the *x*-direction during its third rotation, sampled at a rate proportionate to the spindle speed.

**TABLE 1:** Milling time-domain simulation parameters.

Parameter type	Parameter	Dataset 1	Dataset 2	Dataset 3
Machining parameters	Chip width *b*	0.004 to 0.005	—	—
	Spindle speed *ω*	3,000 to 4,000	—	—
	Feed rate *f*	10.2	—	—
Process-dependent	Number of cutting teeth *N*_*t*_	3	—	—
parameters	Start angle of cut *ϕ*_*s*_	126.9	—	—
	Exit angle of cut *ϕ*_*e*_	180	—	—
	Process dependent coefficient *K*_*s*_	2250e6	1950e6	1950e6
	Force angle *β*	75	—	—
	*x* direction dynamics parameter *k*_*x*_	9e6	—	7e6
	*x* direction dynamics parameter *ζ*_*x*_	0.02	—	—
	*y* direction dynamics parameter *k*_*y*_	1e7	—	1.3e7
	*y* direction dynamics parameter *ζ*_*y*_	0.01	—	—
Simulation parameters	Steps per revolution	256	—	—

Each combination of 200 linearly spaced chip width and 200 spindle speed parameter values in the given ranges yields a signal sample, resulting in a total of 40,000 signal samples in the dataset. The only pre-processing done to this data is mean and standard deviation normalisation, which is done individually for each of the process parameters as well as for the time-domain signals. The validation dataset, which includes 40,000 samples, is created using the same method but with the process parameters moved half a step, i.e. chip width ranging from 0.004025 to 0.005025 and spindle speed from 3,002.5 to 4,002.5. All the datasets are freely accessible on GitLab at https://gitlab.com/EZotoff/cyclestylegan-based-knowledge-transfer-for-a-machining-digital-twin.

### 2.2 Model Architectures

#### 2.2.1 Conditional StyleGAN

**FIGURE 3:**
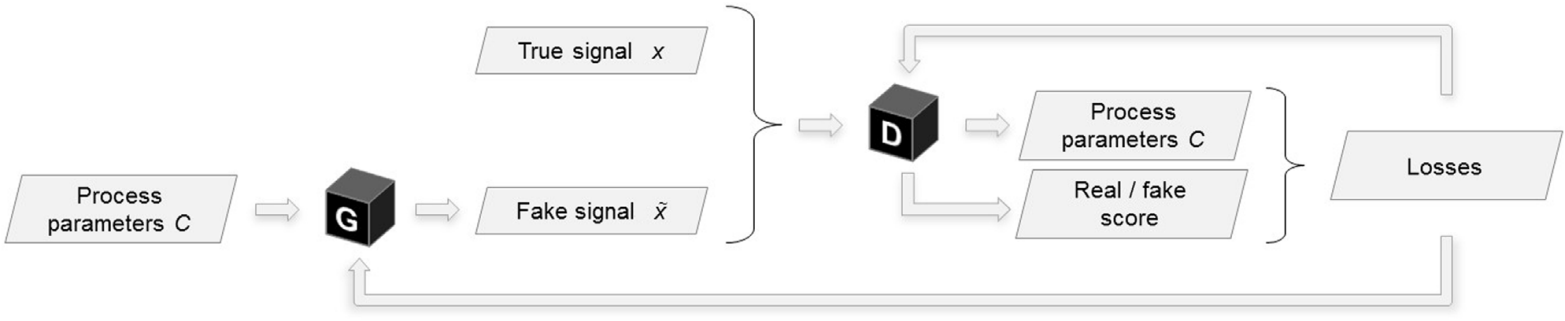
Conditional GAN architecture. G denotes the generator network, D—the discriminator.

**FIGURE 4:**
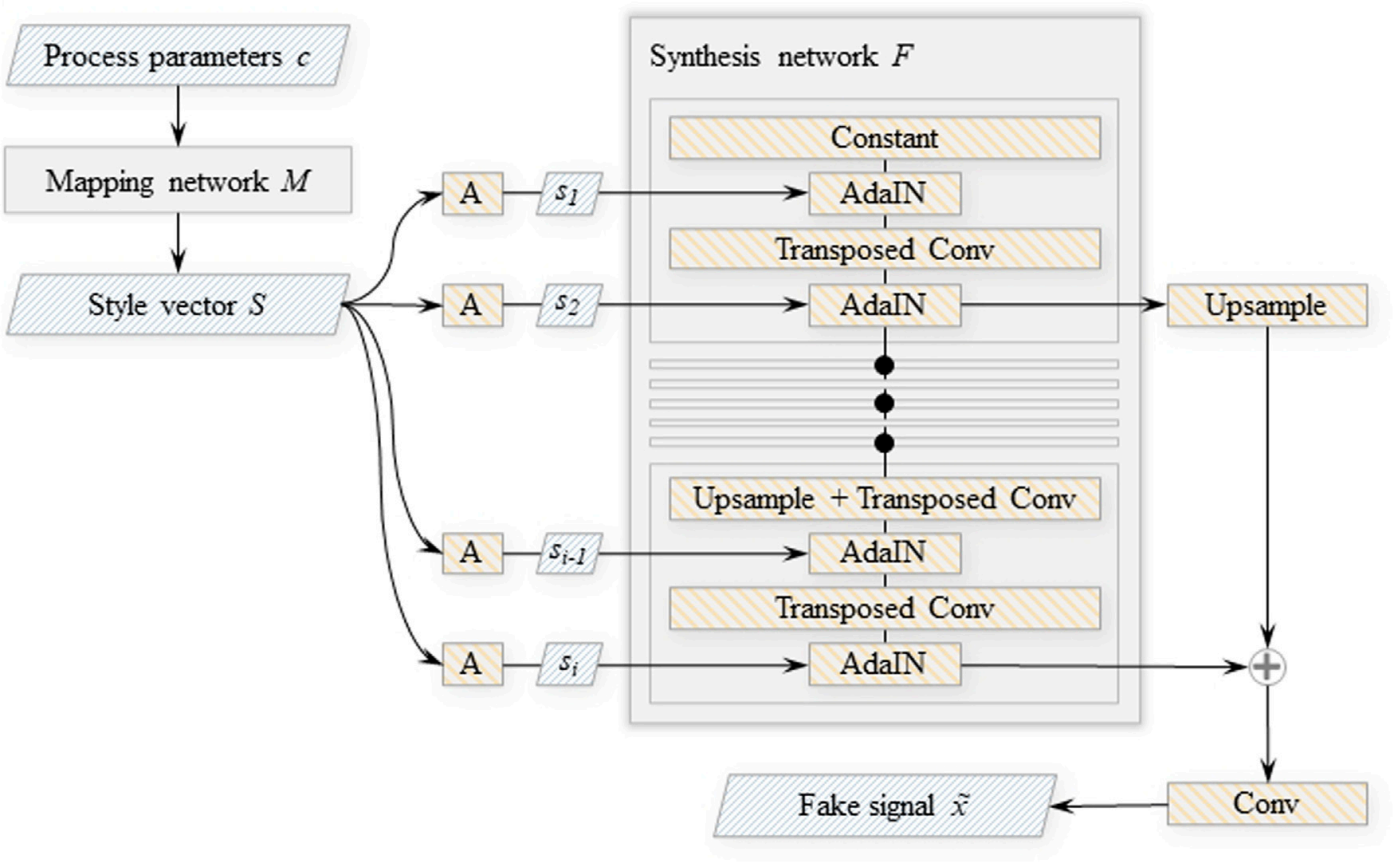
Architecture of the StyleGAN generator network. “A″ denotes learned affine transformations of style components *s*_*i*_; “AdaIN”—adaptive instance normalisation (Huang and Belongie, 2017), outputs of which are modulated by the transformed style components.

StyleGAN (Karras et al., 2018), an image generating model based on two-dimensional deep convolutional networks with the generator enhanced by style-injection inspired by style transfer research works, influenced the digital twin component architecture described in this article. StyleGAN elements are reused for the 1D case of a time-domain signal (Zotov et al., 2020). Because the variation of outputs of the target distribution is deterministic with respect to the input process parameters implies that the training data contains only a single sample per unique label pair, the noise inputs and the mixing regularisation (regularisation applied during training that randomly mixes the disentangled latent with another to produce a sample from the generator *G*) proposed in the StyleGAN paper are excluded from our model. The architecture is improved via the replacement of the random input latent vector with continuous labels *C*, i.e. the machining process parameters: chip width and spindle speed. As shown in Figure 3, the process parameters are utilised as inputs to the generator and as outputs of the discriminator, thus the discriminator learns to not only recognise fake data samples, but also to estimate the labels associated with a particular time-series. A non-linear mapping network *M* projects process parameter inputs into a disentangled latent space. The styles *S* = *M* (*C*) generated by the mapping network from the input labels *C* govern the modulation of outputs of the transposed convolutional layers inside the generator’s synthesis network *F* (Figure 4).

The mapping network *M* is a multi-layer perceptron that has 8 layers, each with 32 neurons that implement leaky ReLU activation functions. The input process parameters *C* are translated into style vectors *S* of length 256 by *M*. A learned constant vector is the initial input to the synthesis network. Multiple blocks, each having two transposed convolutional layers with a convolutional kernel of size 7, consecutively process this learned constant. The output of each block is also upscaled and passed to the output layer, skipping the rest of the convolutional processing. With the exception of the first block, where the constant vector replaces the output of the first convolutional layer, the first convolutional layer in each block upscales the signal length by a factor of two and reduces the number of filters by a factor of two until the number of filters reaches 8. Within the adaptive instance normalisation (AdaIN) operation (Huang and Belongie, 2017) the signals are routed through a leaky ReLU activation function after each convolutional layer, then normalised and blended with the appropriate style component vector. This operation is defined as

$$\text{AdaIN}\left(x_{f},s_{i}\right)=s_{i}^{s}\frac{x_{f}-\mu \left(x_{f}\right)}{\sigma \left(x_{f}\right)}+s_{i}^{b}\text{,}$$ (1)

where $s_{i}^{s}$ and $s_{i}^{b}$ are the scaling and bias components of the style vector at level *i*, and *x*_*f*_ is a filter response that is each normalised individually. The synthesis network’s final layer uses a convolution operation with kernel size *ch* to combine the summed outputs received from the skip connections of the convolutional blocks into a signal with *ch* channels, with *ch* = 1 in our case of displacement along one axis. The discriminator is built as a convolutional neural network composed of several residual blocks. Each block contains two convolutional layers, followed by a downsampling layer. For the parametrisation of the described networks please refer to section 2.3.

The GAN loss function is based on Wasserstein GAN with gradient penalty (WGAN-GP) (Gulrajani et al., 2017). WGAN-GP losses for the generator and the discriminator are

$$\begin{array}{cc} L_{G}^{wgan-gp} & =-\underset{\tilde{x}∼P_{g}}{E}\left[D\left(\tilde{x}\right)\right]\text{,}\\ L_{D}^{wgan-gp} & =\underset{\tilde{x}∼P_{g}}{E}\left[D\left(\tilde{x}\right)\right]-\underset{x∼P_{r}}{E}\left[D\left(x\right)\right]+\lambda_{gp}L^{gp} \end{array}$$ (2)

respectively, where

$$L^{gp}=\underset{\tilde{x}∼P_{\tilde{x}}}{E}\left[{\left(\|\nabla_{\tilde{x}}D\left(\tilde{x}\right){\|}_{2}-1\right)}^{2}\right]$$ (3)

is the gradient penalty, *λ*_*gp*_ is its scaling hyperparameter, *D* is the discriminator network, *x* and $\tilde{x}$ denote the real and fake signals respectively, $P_{r}$ and $P_{g}$ are the real and the generator signal distributions. $P_{\tilde{x}}$ is a distribution sampled uniformly from straight lines between pairs of points from $P_{r}$ and $P_{g}$ (Gulrajani et al., 2017).

The loss functions are adjusted to accommodate the inclusion of machining process parameters in the networks architecture by addition of terms that penalise inaccurate label predictions. This is similar to the approach followed by the authors of InfoGAN (Chen et al., 2016), with the following difference. The accuracy of label predictions for training data $L_{D}^{info}$ impacts only the discriminator, while the accuracy of the predictions for fake data samples $L_{G}^{info}$ is taken into account only by the generator. The loss terms are

$$\begin{array}{cc} L_{G}^{info} & =\frac{1}{n}\overset{n}{\underset{k=1}{\sum }}|c_{k}-{\tilde{c}}_{k,fake}|\text{,}\\ L_{D}^{info} & =\frac{1}{n}\overset{n}{\underset{k=1}{\sum }}|c_{k}-{\tilde{c}}_{k,real}|\text{,} \end{array}$$ (4)

where ${\tilde{c}}_{k,fake}=D\left(\tilde{x}\right)$ is a value of parameter *k* predicted by the discriminator based on a fake signal, ${\tilde{c}}_{k,real}=D\left(x\right)$ is a value predicted from a real signal, and *c*_*k*_ are the true parameter values.

On one hand, the generator is constrained to encode the label information that is identifiable within the synthesised signals. The discriminator, on the other hand, learns the link between labels and samples solely on real data, retaining the non-cooperative aspect of the generator-discriminator minimax game. The total loss functions for the generator *L*_*G*_ and the discriminator *L*_*D*_ are as follows, with *λ*_*info*_ representing the scaling factor for the label prediction accuracy error loss:

$$\begin{array}{c} L_{G}=L_{G}^{wgan-gp}+\lambda_{info}L_{G}^{info}\text{,}\\ L_{D}=L_{D}^{wgan-gp}+\lambda_{info}L_{D}^{info}\text{.} \end{array}$$ (5)

#### 2.2.2 CycleStyleGAN

For the time-series domain adaptation experiment discussed in this paper, the neural network described in section 2.2.1 is extended with elements of CycleGAN (Zhu et al., 2017), an image-to-image translation network that utilises mirrored duplex-GAN architecture for image style transfer between two domains. The underlying intent is the training of two generators: one, *G*_*ab*_, that translates data samples from domain *a* to domain *b* and the other, *G*_*ba*_, that executes the inverse transformation.

The key invention of Zhu et al. (2017), which necessitates the addition of the second GAN structure, is the cycle consistency loss that enforces the equivalence between the true signals from one of the domains $\tilde{x}$ and the reconstructed signals $\tilde{\tilde{x}}$ that are obtained after passing the true signals through both generators, i.e. $\overset{\approx }{x_{a}}=G_{ba}\left(\tilde{x_{b}}\right)=G_{ba}\left(G_{ab}\left(x_{a}\right)\right)$ and $\overset{\approx }{x_{b}}=G_{ab}\left(\tilde{x_{a}}\right)=G_{ab}\left(G_{ba}\left(x_{b}\right)\right)$ . This loss is calculated for both domains *a* and *b* and is defined as:

$$L^{cycle}=|x_{a}-\overset{\approx }{x_{a}}|+|x_{b}-\overset{\approx }{x_{b}}|\text{.}$$ (6)

**FIGURE 5:**
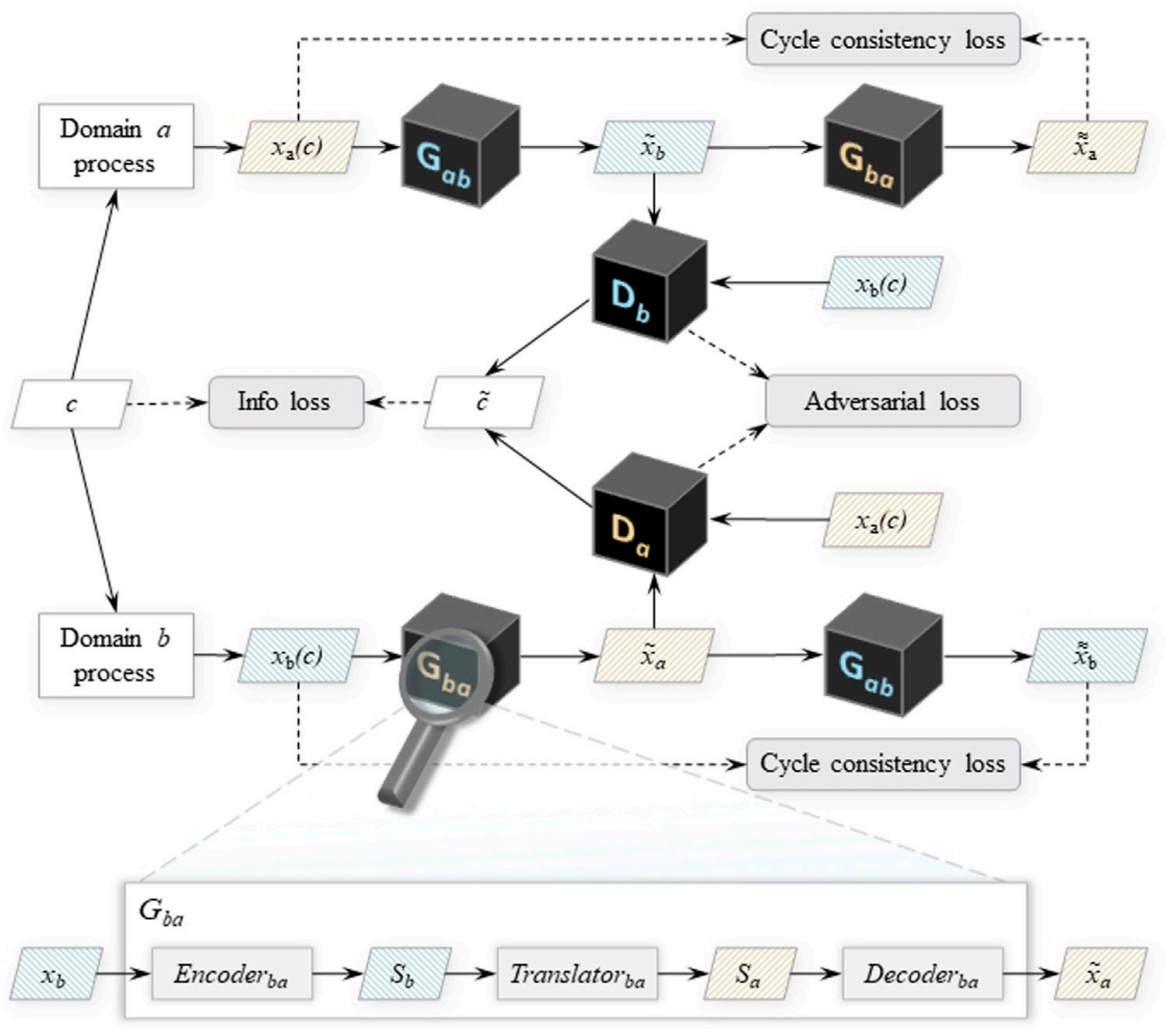
Conditional CycleStyleGAN architecture. *G*_*ab*_ and *G*_*ba*_ denote the generator networks that translate signals from domain *a* to domain *b* and vice versa. *D*_*a*_ and *D*_*b*_ are the discriminators that process domain *a* and *b* signals, both real and the ones translated into their respective domain. The callout on the bottom shows the subnetworks of *G*, using *G*_*ba*_ as the example. Other notation: *c*, $\tilde{c}$ —real and estimated process parameter values; *x*_*a*_ (*x*_*b*_)—true domain *a* (*b*) signals; ${\tilde{x}}_{a}$ ( ${\tilde{x}}_{b}$ ) - signals synthesised *via* translation to domain *a* (*b*); $\overset{\approx }{x_{a}}$ ( $\overset{\approx }{x_{b}}$ )—signals synthesised *via* reconstruction back to domain *a* (*b*); *S*_*a*_ (*S*_*b*_)—style encoding of a domain *a* (*b*) signal. The different colour coding indicates the data structures and the neural networks associated with each domain.

Following the style-based modelling approach used for vibration signal synthesis, the proposed CycleStyleGAN generators also operate on the style encodings of the signals. The generators *G*_*ab*_ and *G*_*ba*_ are thus built as ensembles of three subnetworks: the encoder, the translator and the decoder, and implement the signal translation functions *G*_*ab*_: *x*_*a*_ → *x*_*b*_, *G*_*ba*_: *x*_*b*_ → *x*_*a*_. The encoder network compresses the input signals into their style representations (*Encoder*_*ab*_: *x*_*a*_ → *S*_*a*_ and *Encoder*_*ba*_: *x*_*b*_ → *S*_*b*_), which are then passed onto the translator module. This module transforms the received style vector into the target domain style (*Translator*_*ab*_: *S*_*a*_ → *S*_*b*_, *Translator*_*ba*_: *S*_*b*_ → *S*_*a*_). Finally, the decoder subnetwork synthesises the target domain signal from the translated (*Decoder*_*ab*_: *S*_*b*_ → *x*_*b*_, *Decoder*_*ba*_: *S*_*a*_ → *x*_*a*_). The schematic depiction of the CycleStyleGAN architecture is displayed on Figure 5.

The current work implements the encoder and the translator networks as deep convolutional networks with residual blocks that each contain two convolutional layers. The decoder subnetwork is equivalent to the synthesis network described in section 2.2.1 and is a deep transposed convolutional network with skip connections (see the synthesis network *F* block on Figure 4).

The two discriminators in the CycleStyleGAN model play roles similar to the ones seen in the Conditional StyleGAN model with a key difference in their classification task: the networks now aim to identify whether the signals passed to them belong to their respective domains. The adversarial losses are then formulated as:

$$\begin{array}{cc} L_{G_{ab}}^{wgan-gp} & =-\underset{{\tilde{x}}_{b}∼P_{b}}{E}\left[D_{b}\left({\tilde{x}}_{b}\right)\right]\text{,}\\ L_{G_{ba}}^{wgan-gp} & =-\underset{{\tilde{x}}_{a}∼P_{a}}{E}\left[D_{a}\left({\tilde{x}}_{a}\right)\right]\text{,}\\ L_{D_{b}}^{wgan-gp} & =\underset{{\tilde{x}}_{b}∼P_{b}}{E}\left[D\left({\tilde{x}}_{b}\right)\right]-\underset{x_{a}∼P_{a}}{E}\left[D\left(x_{a}\right)\right]+\lambda_{gp}L_{b}^{gp}\text{,}\\ L_{D_{a}}^{wgan-gp} & =\underset{{\tilde{x}}_{a}∼P_{a}}{E}\left[D\left({\tilde{x}}_{a}\right)\right]-\underset{x_{b}∼P_{b}}{E}\left[D\left(x_{b}\right)\right]+\lambda_{gp}L_{a}^{gp}\text{,} \end{array}$$ (7)

where

$$L_{a}^{gp}=\underset{{\tilde{x}}_{a}∼P_{{\tilde{x}}_{a}}}{E}\left[{\left(\|\nabla_{{\tilde{x}}_{a}}D\left({\tilde{x}}_{a}\right){\|}_{2}-1\right)}^{2}\right]\text{ and}L_{b}^{gp}=\underset{{\tilde{x}}_{b}∼P_{{\tilde{x}}_{b}}}{E}\left[{\left(\|\nabla_{{\tilde{x}}_{b}}D\left({\tilde{x}}_{b}\right){\|}_{2}-1\right)}^{2}\right]$$ (8)

are the gradient penalty terms, *λ*_*gp*_ is the gradient penalty scaling hyperparameter, *D*_*a*_ and *D*_*b*_ are the discriminator networks operating on domain *a* and *b* signals respectively, *x*_*a*_ (*x*_*b*_) and ${\tilde{x}}_{a}$ ( ${\tilde{x}}_{b}$ ) denote the real domain *a* (*b*) signals and the signals translated to the domain *a* (*b*) respectively and $P_{a}$ ( $P_{b}$ ) is the domain *a* (*b*) signal distributions.

As in the case of the information loss formulated for the Conditional StyleGAN, the generators are incentivised to preserve the label information in the synthesised signals, while the discriminators are penalised for misreading the labels encoded within the training data samples. These targets are implemented as the following loss function for the CycleStyleGAN networks:

$$\begin{array}{c} L_{G_{ab}}^{info}=\frac{1}{n}\overset{n}{\underset{k=1}{\sum }}|c_{k}-{\tilde{c}}_{k,b,fake}|\text{,}\;L_{D_{b}}^{info}=\frac{1}{n}\overset{n}{\underset{k=1}{\sum }}|c_{k}-{\tilde{c}}_{k,b,real}|\text{,}\\ L_{G_{ba}}^{info}=\frac{1}{n}\overset{n}{\underset{k=1}{\sum }}|c_{k}-{\tilde{c}}_{k,a,fake}|\text{,}\;L_{D_{a}}^{info}=\frac{1}{n}\overset{n}{\underset{k=1}{\sum }}|c_{k}-{\tilde{c}}_{k,a,real}|\text{,} \end{array}$$ (9)

where ${\tilde{c}}_{k,a,fake}=D_{a}\left({\tilde{x}}_{a}\right)$ and ${\tilde{c}}_{k,b,fake}=D_{b}\left({\tilde{x}}_{b}\right)$ are the values of parameter *k* predicted by the respective discriminators based on signals translated to domain *a* or *b*. ${\tilde{c}}_{k,a,real}=D_{a}\left(x_{a}\right)$ and ${\tilde{c}}_{k,b,real}=D_{b}\left(x_{b}\right)$ are the values predicted from a domain *a* or a domain *b* real signal, and *c*_*k*_ are the true parameter values.

The total losses the generator and the discriminator networks are as follows:

$$\begin{array}{cc} L_{G_{ab}} & =L_{G_{ab}}^{wgan-gp}+\lambda_{info}L_{G_{ab}}^{info}+\lambda_{cycle}L^{cycle}\text{,}\\ L_{G_{ba}} & =L_{G_{ba}}^{wgan-gp}+\lambda_{info}L_{G_{ba}}^{info}+\lambda_{cycle}L^{cycle}\text{,}\\ L_{D_{b}} & =L_{D_{b}}^{wgan-gp}+\lambda_{info}L_{D_{b}}^{info}\text{,}\\ L_{D_{a}} & =L_{D_{a}}^{wgan-gp}+\lambda_{info}L_{D_{a}}^{info}\text{,} \end{array}$$ (10)

Where *λ*_*info*_ represents the scaling factor for the label prediction error loss, *λ*_*cycle*_ is the cycle consistency loss multiplier. *λ*_*info*_ = 10, *λ*_*gp*_ = 10 and *λ*_*cycle*_ = 10 are used to parameterise the network losses during training.

### 2.3 Hyperparameter Optimisation

The neural networks trained during the experiment described in this paper have several hyperparameters that configure their internal structure. The description of what these hyperparameters are and how they are optimised to improve the performance of the models is given below. Where possible, reasonable constraints are enforced to maintain the tractability of the hyperparameter search given the available computational resources.

The number of convolutional blocks, the structure of which is described in section 2.2.1, and number of convolutional filters used in each block are the main hyperparameters that determine the size and complexity of the neural subnetworks. The generative subnetworks, i.e. the synthesis network of the StyleGAN and the decoder of the CycleStyleGAN, receive the number of filters equal to the respective hyperparameter value at the initial convolutional block, that is then downscaled after each block by the filter scaling factor. The minimal number of filters that a block can have is defined via a hyperparameter. The length of the signal is upscaled at the end of each convolutional block, in a way such that the final output signal is of the target length 256. The subnetworks that process the signal in the opposite direction, the discriminators and the CycleStyleGAN encoder, are built in a reverse manner. The number of filters at the last block is determined by the hyperparameter, and this number decreases towards the beginning of the network, while the length of the signal is downscaled after each block from the input’s 256. The full list of the hyperparameters is presented in Table 2.

**TABLE 2:** Model hyperparameters.

Parameter type	Allowed values	Used value
Optimiser		
Generator optimiser type	SGD, Adam	Adam
Discriminator optimiser type	SGD, Adam	Adam
Generator learning rate	0.01, 0.001, 0.0001	0.001
Discriminator learning rate	0.001, 0.0001, 0.00001	0.0001
Conditional StyleGAN generator		
Mapping network *M*		
Number of layers	8	8
Neurons per layer	32	32
Style vector size	32, 128, 256, 1,024	512
Synthesis network *F*		
Number of convolutional blocks	7, 5, 2	2
Starting number of convolutional filters	64, 256, 1,024	256
Filter number scaling factor	2, 4	2
Minimal filters number	8, 64, 128	8
Conditional StyleGAN Discriminator		
Number of convolutional blocks	7, 5, 2	5
Final number of convolutional filters	64, 256, 1,024	64
Filter number scaling factor	2, 4	2
Maximal filters number	128, 512, 1,024	512
CycleStyleGAN generator		
Encoder		
Number of convolutional blocks	7, 5, 2	5
Final number of convolutional filters	64, 256, 1,024	64
Filter number scaling factor	2, 4	2
Maximal filters number	128, 512, 1,024	512
Style vector size	32, 128, 256, 1,024	512
Translator		
Number of convolutional blocks	5, 11	5
Number of filters	2, 32, 128	2
Decoder		
Number of convolutional blocks	7, 5, 2	2
Starting number of convolutional filters	64, 256, 1,024	256
Filter number scaling factor	2, 4	2
Minimal filters number	8, 64, 128	8
CycleStyleGAN Discriminator		
Number of convolutional blocks	7, 5, 2	5
Final number of convolutional filters	64, 256, 1,024	64
Filter number scaling factor	2, 4	2
Maximal filters number	128, 512, 1,024	512

The size of StyleGAN mapping network, which is a feedforward network with fully connected layers, is configured via its depth (the number of layers) and breadth (number of neurons per layer). These are fixed at 8 and 32 respectively, following the StyleGAN work (Karras et al., 2017). The hyperparameters in GAN subnetworks with similar functions are jointly optimised, i.e. the same value is kept between the instances of such hyperparameters in the different networks. Following this approach, the sizes of the style vector output by the mapping network of the StyleGAN model and by the encoder of the CycleStyleGAN are linked. The equivalence of the StyleGAN synthesis and the CycleStyleGAN decoder network architectures is also maintained this way, as well the configurations of both discriminators and the CycleStyleGAN encoder. Another hyperparameter search space constraint adopted from previous experiments sets the discriminator optimiser learning rate to the value of one 10th of the learning rate of the generator.

For further optimisation of the hyperparameter search space we start with only the 12 StyleGAN and optimiser hyperparameters, using a single hyperparameter that defines both learning rates as described above. The StyleGAN model is optimised based on dataset 1 using the Hyperband algorithm (Li et al., 2017). This approach implies training many differently parametrised neural network for a few epochs, selection of the subset of best performing ones with their subsequent further training. After multiple iterations of such selection and training, the hyperparameter values of the best-performing network are considered optimal. After StyleGAN hyperparameter optimisation, the remaining non-linked CycleStyleGAN hyperparameters, which are only the two translator subnetwork hyperparameters, are optimised using the same approach. The hyperparameter optimisation at this stage showed the same results both on dataset 2 and dataset 3.

### 2.4 Training Schedules

The GANs presented in this study are trained until convergence, or until 68,000 000 sample instances are shown to the networks, each instance representing a single time-series picked from the training dataset. The training data instances are fed to the network during training in batches of 1,000 at a time, cycling through all the non-repeating batches. The rate of improvement of the root mean square error (RMSE) metric is measured and averaged over the testing dataset to determine training convergence. Convergence is considered reached if no error reduction is observed over the last 6,800 000 sample instances, i.e. over the last 10% of the maximal total exposure to the training data.

The Conditional StyleGAN model architecture presented in section 2.2.1 is used in two training approaches, for brevity henceforth called retraining and incremental training. Retraining approach implies training of a freshly initialised StyleGAN neural network on a limited set of data from dataset 2 or 3. Incremental training is implemented as a two-stage training schedule. First, a base neural network is trained using all samples available in dataset 1. Second, a neural network initialised with the weights obtained from stage one training (in other words, a copy of the StyleGAN trained on dataset 1) is trained on a given set of samples from datasets 2 or 3. The sets of samples used for training the networks under both approaches are obtained by randomly selecting a fraction of samples from the respective dataset. The percentages of the used samples are 20, 15, 10, 5, 2, 0.8%.

The domain adaptation training of the CycleStyleGANs (see section 2.2.2 for the model architecture) is performed using the full source domain data (i.e., dataset 1) and a subset of the target domain data. The percentages of the used samples are 20, 15, 10, 5, 2, 0.8, 0.2%. The sample sets of datasets 2 and 3 used under this approach are the same as the sets used for incremental training of the StyleGAN described above.

## 3 Results

The analysis presented in this section seeks the validation of the proposed CycleStyleGAN architecture as a knowledge transfer technique under the target domain data scarcity constraint. To this extent, the accuracies of the CycleStyleGAN model instances are compared with the accuracies of the StyleGAN networks, thus presenting the performance of the proposed domain adaptation method against the incremental learning approach. For comparison fairness, the underlying subnetworks of the models are parametrised identically wherever possible, and the models are treated with the same sets of samples during training and are evaluated on the same validation data.

Each trained StyleGAN and CycleStyleGAN model is evaluated by a generative error metric defined as the average of the mean absolute error (MAE) between the target signals from the validation data ( $x\left(\right.c^{val}$ ) and the synthesised signals, i.e. the signals created by StyleGAN from parameters $\tilde{x}=G\left(c^{val}\right)$ or translated by the *a* → *b* CycleStyleGAN from the domain *a* signals $\tilde{x}=G_{ab}\left(x\left(c^{val}\right)\right)$ :

$$\bar{E}=\frac{1}{m}\overset{m}{\underset{j=1}{\sum }}E\left(c_{j}^{val}\right)\text{, where }E\left(c\right)=\frac{1}{n}\overset{n}{\underset{i=1}{\sum }}\left|x_{i}\left(c\right)-{\tilde{x}}_{i}\left(c\right)\right|\text{,}$$ (11)

where *m* is number of samples in the validation dataset and *n* is the signal length. All models are consecutively trained as described in section 2.4, starting with the highest fraction of the target dataset, 20%. The experiment for a particular training approach and dataset is interrupted if the obtained error distribution includes any points above the model deficiency threshold. This threshold is inferred from the generative error evaluated on the target domain validation using the StyleGAN model obtained during stage one of the incremental training, i.e. the model trained only on the source domain dataset:

$${\bar{E}}_{thld}=\frac{1}{m}\overset{m}{\underset{j=1}{\sum }}E_{thld}\left(c_{j}^{val}\right)\text{, where }E_{thld}\left(c\right)=\frac{1}{n}\overset{n}{\underset{i=1}{\sum }}\left|x_{i}^{target}\left(c\right)-{\tilde{x}}_{i}^{source}\left(c\right)\right|\text{,}$$ (12)

For the sake of limiting the computations required to execute the experiment, and considering that the focus of this work is on the transfer learning with minimal amount of available data, we do not evaluate the models using more than 20% of the target domain data. The error levels of all models differ insignificantly for training runs utilising 15% or more data. The errors are averaged across multiple training runs for each combination of samples.

**FIGURE 6:**
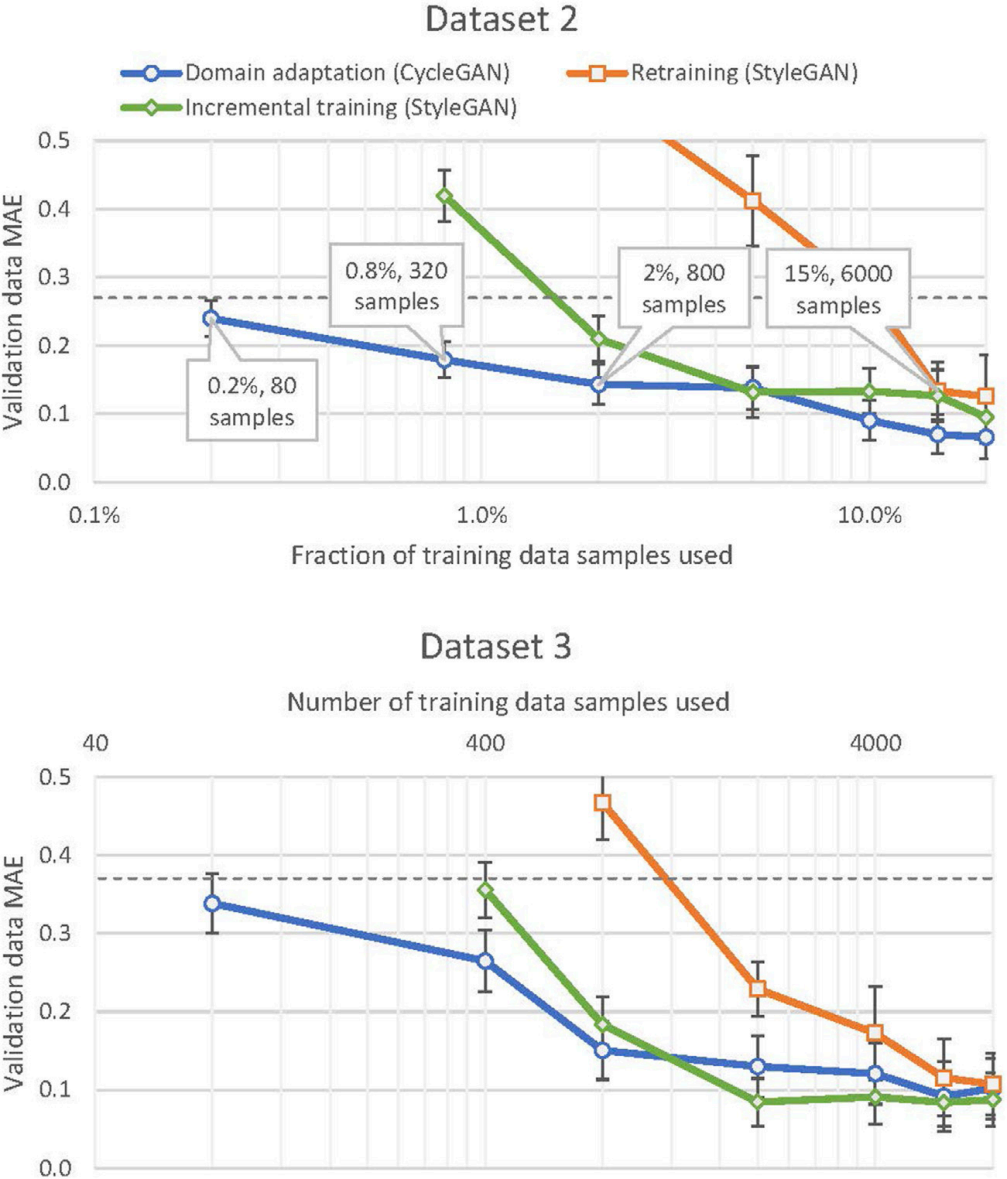
Model error (*Y*-axis) plotted against the amount of data (*X*-axis, log scale) used for training the networks under the three approaches, separated by target domain dataset. The vertical error bars indicate the standard deviation of the model error across several runs under the same conditions. The dashed horizontal lines on both subcharts represent the errors ${\bar{E}}_{thld}$ of the models trained on dataset 1 when evaluated against dataset 2 and 3 validation data respectively.

The training performance distributions of the models at the different levels of target domain data limitations are presented on Figure 6. Dataset 2 represents a scenario of small difference between the source and the target domains, e.g. as a result of minor variations in material characteristics or environment conditions. Dataset 3 expresses a case of significantly different characteristics underlying the target domain signals, for example arising from a machining tool with a different geometry. A model trained without any source domain knowledge performs well on both datasets when trained using 6,000 (out of the total 40,000) or more target domain training data samples, with smaller training dataset size leading to a sharp drop in the models’ performance. The incremental learning approach shows a similar pattern of severe generative accuracy degradation, but below a more strict data limitation constraint: 2,000 samples. The domain adaptation implementation using the CycleStyleGAN architecture proposed in this paper displays different behaviour to the aforementioned approaches. The quality of the generated signals significantly degrades only when trained on less than 800 target domain data samples, and the degradation below this point is smoothly approaching the ${\bar{E}}_{thld}$ error level.

These results imply that the CycleStyleGAN error has an upper bound at ${\bar{E}}_{thld}$ , the target domain accuracy level of the model trained only on the source domain data. Therefore, the reliability of this model can be estimated from the expected difference between the source and the target domains. The use of the source domain data during CycleStyleGAN training ensures that the model does not suffer from catastrophic forgetting and does not overfit to the small subset of the observed data samples, contrary to what happens to the neural networks trained from scratch or trained incrementally. The CycleStyleGAN domain adaptation is thus potentially usable with any amount of data available at hand at a given moment and can be expected to reach peak performance with the amount of target domain data one order of magnitude lower than a model trained from scratch. For an industrial implementation this means that, on one hand, the value of the knowledge extracted from the source model is not diluted during the knowledge transfer process, and, on the other hand, that the adaptation of the transferred information to a new process can be initiated along with the launch of this process. For a process that requires generative accuracy above the threshold bound, the proposed method enables a reduction of the pre-launch data acquisition effort almost tenfold.

The available computational power limitations implied that the number of training repetitions in the described experiment had to be limited to three for each set of training conditions. While this simplification blurs the precision of the estimated model error distributions, the variation in the model’s effectiveness provides sufficient evidence to support the claims presented in the current work.

## 4 Discussion

While GAN-based approaches are widely popular in the image processing domain adaptation domain (Liu and Tuzel, 2016; Bejiga and Melgani, 2018; Hoffman et al., 2018; Feng et al., 2020; Shahbazi et al., 2021; Tan et al., 2021), their implementation for time-series generation problems is relatively limited. Several use cases are described in the energy output prediction (Chen et al., 2018), in music (Mogren, 2016), and in medical (Esteban et al., 2017; Tseng et al., 2017) and manufacturing (Wang Z. et al., 2018) time-series generation.

The research focus on GANs in manufacturing is presently dominated by data augmentation aimed at supporting a main classification model (Han et al., 2019; Wang et al., 2019b,a). This is also confirmed by the reviews on manufacturing applications of artificial neural networks, where data augmentation is identified as the sole use case for GAN models (Wang J. et al., 2018; Kusiak, 2019; Qi et al., 2019; Jiao et al., 2020). An increasing plethora of works is being published in the recent years that propose solutions that enhance the classification accuracy of machinery fault detection, as evidenced in the review papers covering just the deep learning research on this topic (Li et al., 2020). Research on domain adaptation approaches in manufacturing follows the same pattern (Zheng et al., 2019). Manufacturing applications of GANs as a primary generative instrument can be found in fields of sampling resolution enhancement (Alawieh et al., 2019) and generative design of material structures (Tan et al., 2019).

To the best of authors knowledge, the research on the generative function of GANs for manufacturing process simulation is largely unexplored. Even less studied area is the application of digital twin simulations in the context of knowledge extraction for Industry 4.0.

These research gaps are addressed within the work described in this paper. The style-based representation of the simulated vibration signals has been shown in a previous publication to be useful both for performance improvement and for analysis of the underlying model (Zotov et al., 2021). Building on the GAN extensions for the transfer learning tasks, namely the CycleGAN architecture, this paper introduces the style features into the domain adaptation model via the novel CycleStyleGAN architecture and proposes the following Industry 4.0 use case for the resulting knowledge transfer tool.

A physics-based model for a fleet of machines would be the same in a real-world situation, but each unit would be handled under various circumstances and have somewhat varied characteristics. Configuration of the physics-based models for a specific instance depends on the particular process conditions and is generally a labour-intensive process, which thus becomes exponentially costly when employed on a broad scale. While a physics-based model could serve as a component of a digital twin simulation, it would inevitably simplify and idealise the process, discarding individual diversity of environmental and dynamic factors influencing the manufacturing process due to their modelling complexity or computational cost. These complex phenomena are mirrored in real-world process data and may therefore be captured via data-driven model training.

**FIGURE 7:**
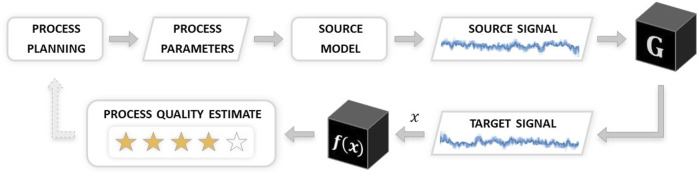
Proposed generator model as a part of a process optimisation flow.

Control over the input machining process parameters guides the process signal synthesis in the proposed CycleStyleGAN model. Therefore, the model may serve as a vibration simulation tool that translates the process parameter inputs into vibration signal outputs when combined with a source domain simulation model that produces source signals from process parameters. Such a simulation may be used for CNC machining process optimisation and planning. Researchers have shown how signal data could be utilised for product quality prediction (He and Wang, 2018; Papananias et al., 2019b; Leco and Kadirkamanathan, 2021), enabling the prediction of manufacturing defects prior to manufacturing. Furthermore, machining process stability estimates may be improved by substituting generative models for parts of the physical data, which is another future study path suggested by machining stability specialists (Greis et al., 2020). The proposed CycleStyleGAN model is thus usable as a process optimisation tool: by probing the model to acquire parameter-signal pairs and evaluating the resultant process quality based on the received signals, an optimisation process loop would seek the optimum within the parameter space. Schematic representation of this process flow is depicted on Figure 7. This paper shows how an established physics-based or data-driven model can be sourced, thus extracting the value of the information contained within the said model for further reuse. We believe the cost-efficiency of the proposed model to be an important driver towards the widespread use of digital twin solutions along the transition to Industry 4.0.

A drawback of the CycleStyleGAN model architecture, as compared to the StyleGAN, is its higher computational resource requirements. The mirrored GAN structure uses approximately double the memory and double the computations during training. While these differences are negligible for a trained model due to the efficiency of the neural networks at inference time, the training process computations are twice as costly. Although the costs of computational hardware are incomparable to the manufacturing process expenses in most cases, certain high-volume low-value production industries might find the computational overhead exceeding the expected value of such simulation model adaptation. Such businesses, having relatively lower data acquisition costs, can be expected to be able to effectively employ machine learning models without the need for transfer learning.

Possible extensions of the proposed domain adaptation approach may consider inter-task transfer learning. For example, the prediction of the machining cutting forces from the vibration signals may useful for downstream process analysis. Another research gap the exploration of which might lead to improved generative performance of the underlying model is the specialised subnetwork architecture. The current literature presents multiple options for structuring of these neural networks, but a comparative analysis of the performance of these architecture choices is yet to reach the science community. Linked to this is the application of sophisticated neural network architecture search approaches. An extension of an advanced method like the ES-HyperNEAT (Risi and Stanley, 2012) might aid not only the hyperparameter optimisation of a pre-defined network structure, but also in discovery of a novel composition of the neural network.

## Data Availability

The datasets presented in this study can be found in online repositories. The names of the repository/repositories and accession number(s) can be found below: https://gitlab.com/EZotoff/cyclestylegan-based-knowledge-transfer-for-a-machining-digital-twin.
